# Nrf2 overexpression increases risk of high tumor mutation burden in acute myeloid leukemia by inhibiting MSH2

**DOI:** 10.1038/s41419-020-03331-x

**Published:** 2021-01-05

**Authors:** Ping Liu, Dan Ma, Ping Wang, Chengyun Pan, Qin Fang, Jishi Wang

**Affiliations:** 1 Department of Hematology, Affiliated Hospital of Guizhou Medical University, Guizhou Province Institute of Hematology, Guizhou Province Laboratory of Hematopoietic Stem Cell Transplantation Centre, 550004 Guiyang, China; 2 Basic Medical College, Guizhou Medical University, 550004 Guiyang, China; 3 Department of Pharmacy, Affiliated Hospital of Guizhou Medical University, 550004 Guiyang, China; 4 National Clinical Research Center for Hematologic Diseases, the First Affiliated Hospital of Soochow University, 215006 Suzhou, China

**Keywords:** Cancer genomics, Apoptosis

## Abstract

Nuclear factor erythroid 2-related factor 2 (Nrf2, also called NFE2L2) plays an important role in cancer chemoresistance. However, little is known about the role of Nrf2 in tumor mutation burden and the effect of Nrf2 in modulating DNA mismatch repair (MMR) gene in acute myeloid leukemia (AML). Here we show that Nrf2 expression is associated with tumor mutation burden in AML. Patients with Nrf2 overexpression had a higher frequency of gene mutation and drug resistance. Nrf2 overexpression protected the AML cells from apoptosis induced by cytarabine in vitro and increased the risk of drug resistance associated with a gene mutation in vivo. Furthermore, Nrf2 overexpression inhibited MutS Homolog 2 (MSH2) protein expression, which caused DNA MMR deficiency. Mechanistically, the inhibition of MSH2 by Nrf2 was in a ROS-independent manner. Further studies showed that an increased activation of JNK/c-Jun signaling in Nrf2 overexpression cells inhibited the expression of the MSH2 protein. Our findings provide evidence that high Nrf2 expression can induce gene instability-dependent drug resistance in AML. This study demonstrates the reason why the high Nrf2 expression leads to the increase of gene mutation frequency in AML, and provides a new strategy for clinical practice.

## Introduction

Acute myeloid leukemia (AML) is a malignant tumor of myeloid progenitor cells characterized by immature myeloid cell proliferation and bone marrow failure. Standard “7 + 3” induction therapy, which combines a nucleoside analogue such as cytarabine (Ara-C) for 7 days with an anthracycline for 3 days, is highly effective in killing leukemic cells in AML. Despite the fact that the majority of AML patients achieve complete remission after chemotherapy, the 5-year overall survival is very poor, especially in patients over 60 years of age^1,2^. Most patients die of their disease due to either refractory (initial resistance to chemotherapy) or relapsed AML^3^. Therefore, the resistance of leukemia cells to chemotherapy drugs becomes the main obstacle in the treatment of AML.

Many hypotheses have been proposed to explain therapeutic resistance in AML, including the persistence of leukemic stem cells^4^, increased antioxidant defense systems^5^, altered expression of drug influx and efflux transporters^6^, evasion of cell death^7^, and epigenetic mechanisms including DNA methylation and histone modification^8,9^. The tumor microenvironment is also involved in the development of acquired resistance to chemotherapeutics^10^. In addition, tumor cells are insensitive to chemotherapeutic drugs, due to the presence of complex abnormal karyotypes of chromosomes and gene mutations^11^. Therefore, exploring the molecular mechanism of gene instability-dependent drug resistance is a significant strategy to overcome the chemoresistance and relapse.

Nuclear factor-erythroid 2-related factor 2 (Nrf2, also called NFE2L2) is one of the transcription factors involved in cancer cell survival pathways and implicated in protecting cancer cells from apoptosis^12^. Nrf2 functions to change the sensitivity of the tumor cell environment to oxidants and electrophiles by stimulating the transcriptional activation of cytoprotective genes^13^. There are many studies showing elevated expression of Nrf2 in various types of tumors such as head and neck^14^, gastric^15^, breast^16^, gallbladder^17^, and ovarian^18^ cancer. Upon oxidative stress, Nrf2 signaling is activated and protects tumor cells from cell death by upregulating reactive oxygen species (ROS) scavenging enzymes that counterbalance production^19^. Nrf2 protects tumor cells from death by cooperating with other pathways, which plays a role in apoptosis regulation. For example, mutant p53 can upregulate Nrf2 expression at the transcriptional level, resulting in anti-apoptosis and chemotherapy resistance^20^. p62 is another Nrf2 target, which upon phosphorylation facilitates Nrf2 translocation to the nucleus, thereby inhibiting apoptosis of cancer cells^21^. Moreover, Nrf2 binds the ARE sequence on its promoter to upregulate the Bcl-2 expression, which prevents cellular apoptosis and induces drug resistance^22^. These studies indicate that Nrf2 inhibits apoptosis and confers resistance to anticancer therapy through different pathways, which play a crucial role in tumor survival and chemoresistance.

However, the existing reports are mostly limited to the effect of high Nrf2 expression on gene instability-independent drug resistance, and there are few reports on Nrf2 participating in the regulation of gene instability-dependent chemoresistance. Genomic instability plays an important role in the development of cancer^23^. The DNA mismatch repair (MMR) is vital for the maintenance of genomic stability of human cells. Biochemical and genetic studies have found several MMR genes, including MSH2, MLH1, MSH6, PMS2, POLD2, RFC4 and so on^24,25^. Defective mismatch repair cells exhibit a higher frequency of mutation in both coding and noncoding microsatellite sequences. MMR deficiency leading to microsatellite instability (MSI) has been recognized as a distinct tumorigenesis pathway^26^. Additionally, DNA repair defects are associated with the development of resistance to chemotherapeutics, in both solid tumors and hematological malignancies^27,28^.

In this study, we sought to investigate the role of Nrf2 in AML gene instability-dependent drug resistance. We found that Nrf2 was significantly upregulated in AML with high tumor mutation burden and chemoresistance. Further analysis revealed that Nrf2 overexpression inhibited MSH2, thereby promoting gene mutant chemoresistance of AML cells both in vitro and in vivo. Mechanistically, the role of Nrf2 in causing DNA MMR deficiency was achieved by regulating JNK/c-Jun signaling.

## Results

### Higher tumor mutation burden in AML patients with Nrf2 overexpression

**Fig. 1:**
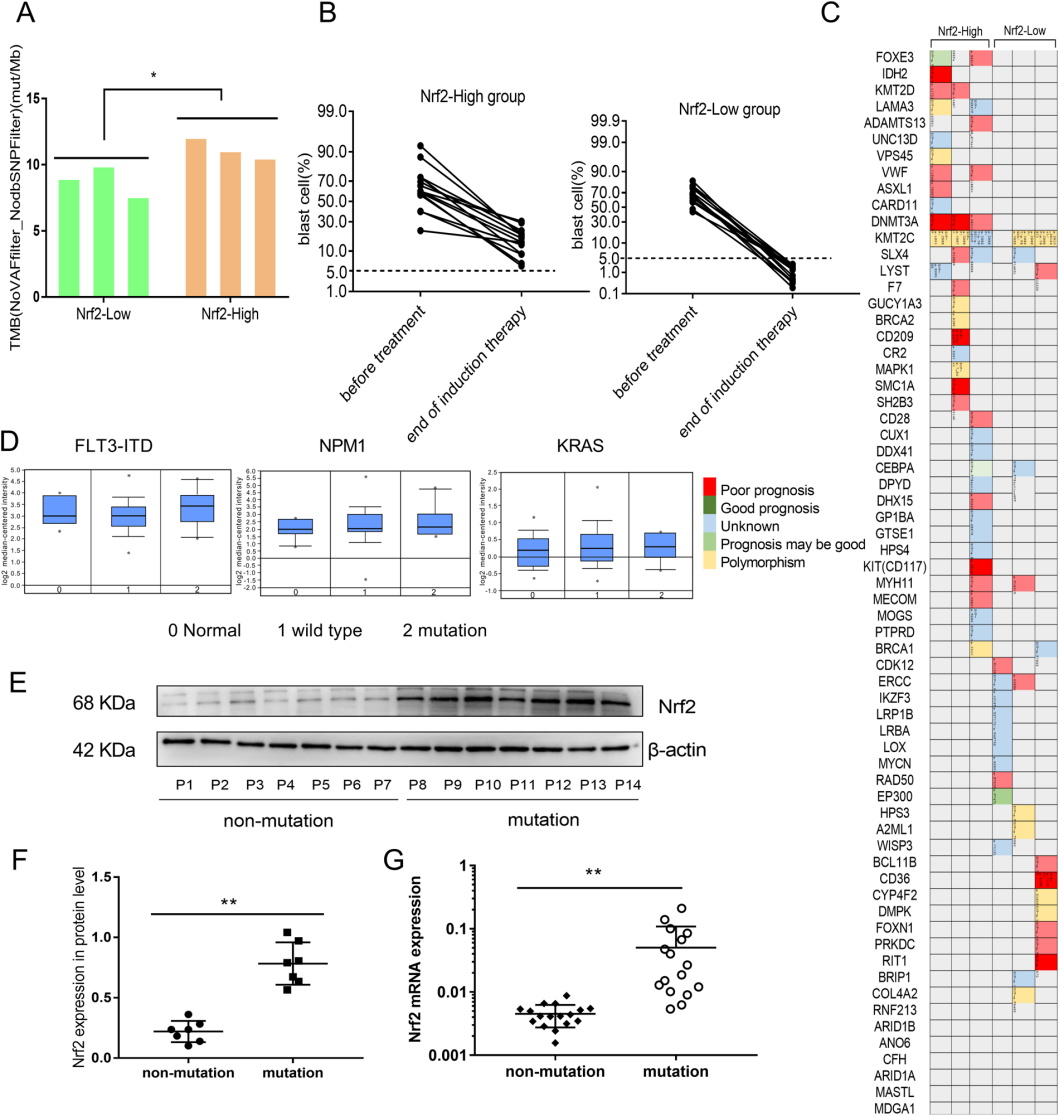
Nrf2 expression and tumor mutation burden in AML. **a** TMB values of mutation sites in Nrf2 high/low expression group (n = 6). **b** The percentage of blast cells were detected in Nrf2 high/low AML specimens (*n* = 30). **c** Disease-related gene mutations were shown in AML patients with high/low expression of Nrf2 by whole-exon sequencing (*n* = 6). **d** Studies in the Oncomine database showed higher mRNA expression of Nrf2 in AML patients with FLT3-ITD, NPM1, KRAS positive mutations (n = 526). **e** Expression levels of Nrf2 protein were detected in AML specimens by western blotting (P: patient, *n* = 14). **f** Quantification of Nrf2 expression in AML samples. **g** mRNA expression of Nrf2 in AML by qRT-PCR (*n* = 33). Results are presented as means ± SD; TMB, tumor mutation burden. *p < 0.05, **p < 0.01.

With the development of the whole-genome sequencing technology, the increase of tumor mutation burden has been found to be one of the important reasons for chemoresistance and relapse in AML^29^. In our study, AML patient specimens were divided into two groups either expressing high or low levels of Nrf2 based on qRT-PCR, using the median Nrf2 expression levels as cut-off values. Then, we used whole-exon sequencing to detect gene mutations and calculated the tumor mutation burden values based on the mutation site. The tumor mutation burden values in the Nrf2-High expression group was significantly higher than that in the Nrf2-Low group (11.21 ± 0.459 mut/Mb vs. 8.82 ± 0.670 mut/Mb (*P* < 0.05)) (Fig. 1a). And disease-related gene mutations were shown in Fig. 1c. In addition, patients in the Nrf2-High group had more blast cells and less remission after standard chemotherapy, which had a higher risk of relapse or drug resistance (Fig. 1b). To investigate the role of Nrf2 in AML patients with gene mutations, we examined the gene expression level of Nrf2 in different types of gene mutations using the Oncomine database (https://www.oncomine.org/). We noticed that the expression of Nrf2 was upregulated in AML patients with FLT3-ITD, NPM1, KRAS positive mutations (Fig. 1d). We further analyzed the Nrf2 protein expression level in non-mutation and mutation AML patients by western blotting. The results showed that the protein level of Nrf2 in the mutation group was higher than that in the non-mutation group (*P* < 0.01, Fig. 1e, f and Fig. S1A–D). Then we compared the mRNA levels of Nrf2 in mutated and non-mutated AML samples. The results showed that the Nrf2 expression in the gene mutated group was significantly higher than that in the non-mutated group (*P* < 0.01, Fig. 1g). Therefore, we preliminarily concluded that high Nrf2 expression in AML was related to the high tumor mutation burden.

### High Nrf2 expression inhibited the DNA mismatch repair pathway in AML

**Fig. 2:**
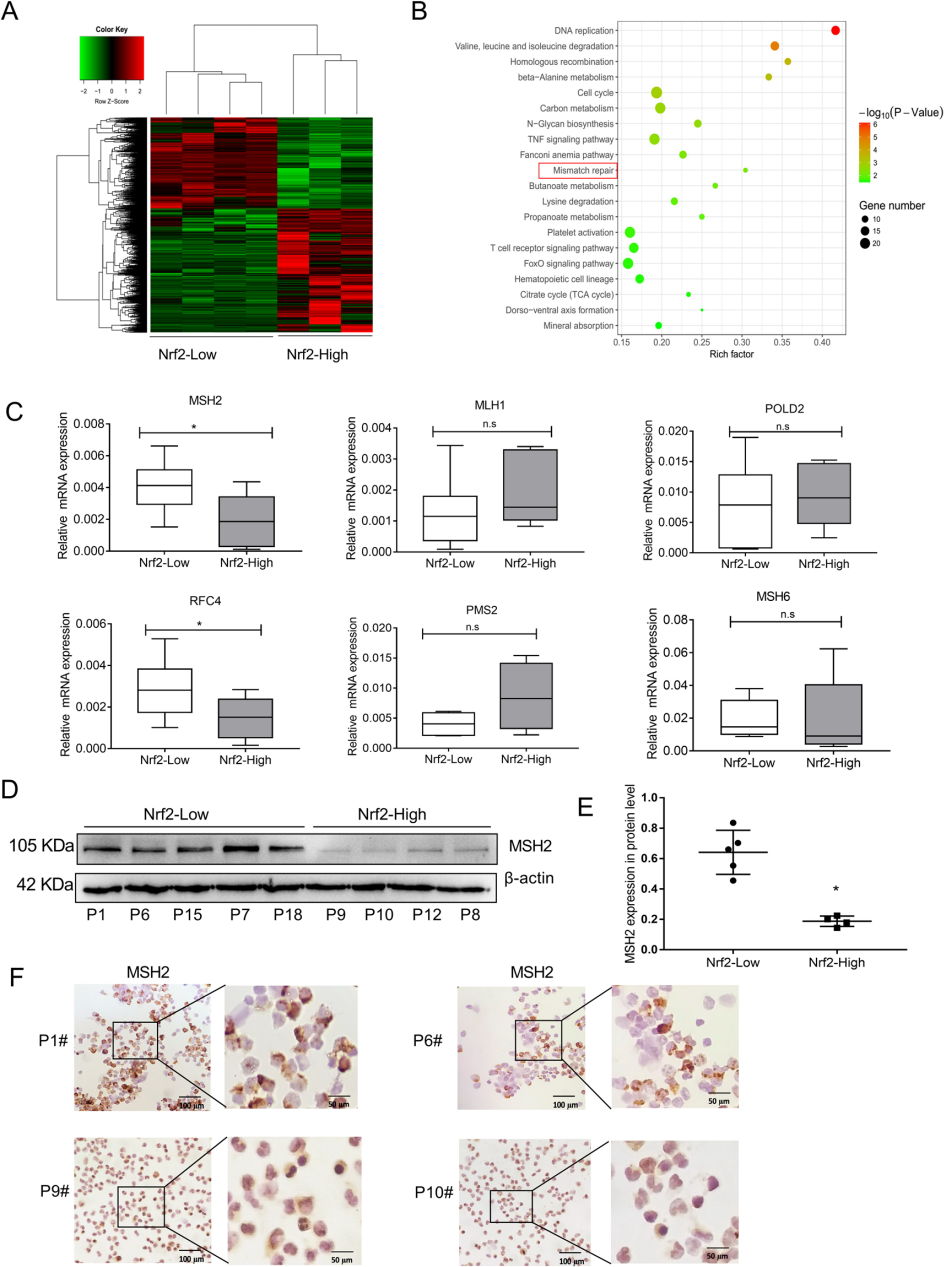
Nrf2 inhibited DNA mismatch repair pathway in AML. **a** The heatmap of hierarchical clustering showed the differentially expressed genes in the Nrf2 high/low expressed group based on RNAseq analysis (*n* = 7). **b** KEGG pathway analysis showed that Nrf2 expression was inhibited DNA Mismatch repair (MMR) in the AML. The KEGG pathway with P < 0.05 was shown in a bubble plot. **c** qRT-PCR analysis of the expression of the MMR genes, including MSH2, MLH1, POLD2, RFC4, PMS2, and MSH6 in the Nrf2 high/low expressed group (*n* = 33). **d** Expression levels of MSH2 protein were detected in AML samples by western blotting (n = 9). **e** Quantification of MSH2 expression in Nrf2-High group and Nrf2-Ligh group. **f** Representative images of ICC staining of MSH2 in AML (P1 and P6, Nrf2-Low group; P9 and P10, Nrf2-High group), Scale bars: 100 and 50 μm from left to right. Results are presented as means ± SD; *p < 0.05, **p < 0.01, ns, no significance.

In order to further understand the molecular mechanism of Nrf2 on the tumor mutation burden rate in AML, we examined transcriptome sequencing (RNAseq) in the above patients, removed the unqualified samples, and analyzed the difference of gene expression (GEO accession: GSE160499). As shown in Fig. 2a, the heatmap illustrated the differentially expressed genes between Nrf2-High and Nrf2-Low group. We then performed KEGG pathway enrichment analysis on the genes differentially expressed in AML samples. The results indicated that the DNA MMR pathway was significantly inhibited in AML with high expression of Nrf2 (Fig. 2b). It is well-known that the MMR pathway is an important way to influence point mutation^30^. Thus, qRT-PCR analysis was employed to identify MMR genes. We found that the mRNA expression of MSH2 in the Nrf2-Low group was significantly higher than that of the Nrf2-High group (*P* < 0.05, Fig. 2c). A similar tendency was observed in the protein level of MSH2 by western blotting. MSH2 was also decreased in Nrf2-High expressing AML patients (*P* < 0.05, Fig. 2d, e). Finally, we performed immunocytochemical (ICC) detection in AML patients with different Nrf2 expression. The ICC staining results showed that patients in the high Nrf2 expression group had a lower level of MSH2 when compared with patients in the Nrf2-Low group (Fig. 2f). These results suggested that high Nrf2 expression inhibited MSH2 in AML.

### High Nrf2 expression increased the resistance of AML cell lines to Ara-C while inhibited the expression of MSH2

**Fig. 3:**
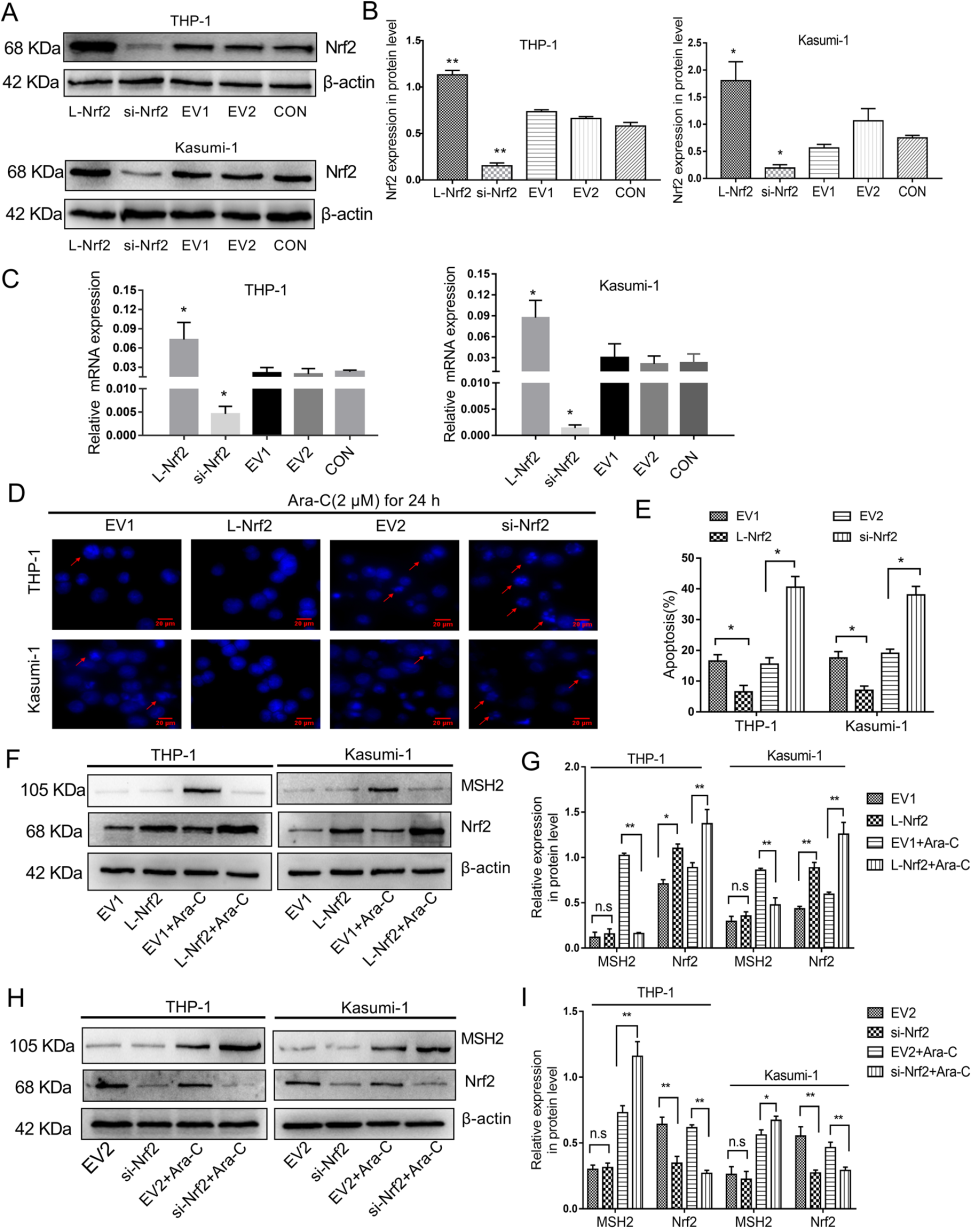
Nrf2 induced Ara-C resistance and suppressed MSH2. **a** Nrf2 was overexpressed or silenced in THP-1 and Kasumi-1 cell lines determined by western blot analyses. **b** The relative gray values were shown in histogram. **c** Nrf2 was overexpressed or silenced in THP-1 and Kasumi-1 cell lines determined by qRT-PCR analyses. **d**, **e** The necrotic cells in different groups were detected after Hoechst 33342 staining (scale bars, 20 µm). **f** The Nrf2-overexpressing cells were treated with or without Ara-C (2 μM) for 24 h. The Nrf2 and MSH2 protein levels were assessed by western blotting. **g** The relative gray values were shown in histogram. **h** The silencing Nrf2 cells were treated with or without Ara-C (2 μM) for 24 h. The Nrf2 and MSH2 protein levels were assessed by western blotting. **i** The relative gray values were shown in histogram. Data are presented as the mean ± SD of three independent experiments. EV, empty vector. Ara-C, cytarabine. *P < 0.05, **P < 0.01, ns, no significance.

According to the above results in clinical samples, we speculated that Nrf2 high expression inhibited MSH2 expression, caused MMR deficiency and increased the tumor mutation burden, which can induce gene instability-dependent drug resistance. To test this hypothesis, we overexpressed and silenced Nrf2 in two different AML cell lines (THP-1 and Kasumi-1). The Nrf2 expression levels in these cell lines were verified by Western blotting and qRT-PCR (Fig. 3a–c). Then, hoechst 33342 stain was applied to evaluate the effect of Nrf2 on nuclear fragmentation (apoptosis marker) in THP-1 and Kasumi-1 cells. The data showed that Nrf2 overexpression in AML cell lines decreased the apoptosis of cells treated with 2 μM Ara-C for 24 h (Fig. 3d, e). Besides, we confirmed that Ara-C led to the accumulation of MSH2 protein in a concentration-dependent manner (Fig. S2A, B). Moreover, we found that Nrf2 overexpression potently decreased the protein level of MSH2, whereas MSH2 protein levels were increased upon Nrf2 downregulation in THP-1 and Kasumi-1 cells (Fig. 3f–i). Therefore, Nrf2 was shown to promote Ara-C resistance in AML cells by inhibiting MSH2.

### AML cells with Nrf2 high expression had a higher risk of drug resistance associated with gene mutation in vivo

**Fig. 4:**
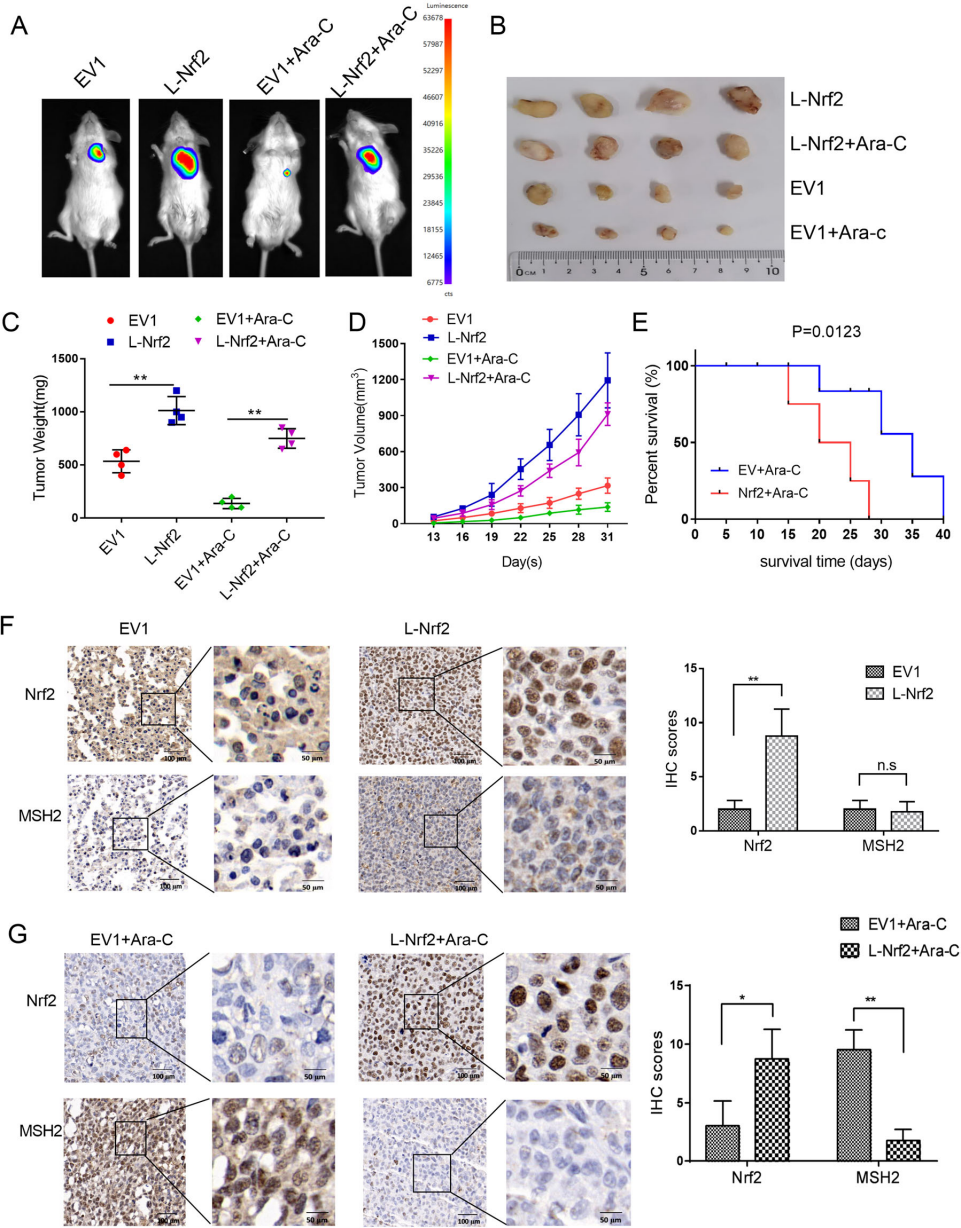
Overexpression of Nrf2 conferred higher risk of mutant drug resistance in vivo. **a** Representative images of tumor-bearing mice in the indicated cells. **b** Images of subcutaneous xenografts from mice in the EV1, L-Nrf2, EV1 + Ara-C, and L-Nrf2+Ara-C groups. *n* = 16. **c** Tumor weight change curves for subcutaneous xenografts. **d** Tumor volume growth curves for subcutaneous xenografts. **e** Survival analysis curves for subcutaneous xenografts. Survival was plotted by using the Kaplan–Meier method. **f** The expression of Nrf2 and MSH2 was examined in xenograft tumor tissue sections using immunohistochemistry (scale bars: 100 and 50 μm from left to right). *P < 0.05, **P < 0.01.

To confirm the effect of Nrf2 in AML cell growth in vivo, the NOD-SCID/IL2Rγc mice xenograft model was established by subcutaneous injection of Nrf2 or empty vector transfected THP-1 cells. And the mice were treated with Ara-C as soon as the tumor became palpable. As shown in Fig. 4a–b, Nrf2 overexpression resulted in a significant increase in tumor growth compared with that in the EV group. Nrf2 overexpression effectively promoted the tumor weights (Fig. 4c) and tumor volumes (Fig. 4d) compared to the EV group (*P* < 0.05). Moreover, treatment with Ara-C resulted in a significant reduction in tumor growth. As shown in Fig. 4e, mice transplanted with L-Nrf2 cells had the shortest survival, whereas mice transplanted with EV cells had a prolonged overall survival (*P* < 0.05). Furthermore, MSH2 expression was examined in paraffin-embedded tumor tissues by an IHC assay. In vivo, there was no significant alterations in MSH2 between the Nrf2 overexpression group and the EV group without Ara-C treatment (Fig. 4f). However, after treatment with Ara-C, MSH2 expression was still weaken in the Nrf2 overexpression group, but increased in the EV group (Fig. 4g). Therefore, these data demonstrated that Nrf2 overexpression promoted tumor growth and inhibited MSH2, which contributed to a higher risk of chemoresistance associated with a gene mutation in vivo.

### The high expression of Nrf2 inhibited MSH2 in a ROS-independent manner

**Fig. 5:**
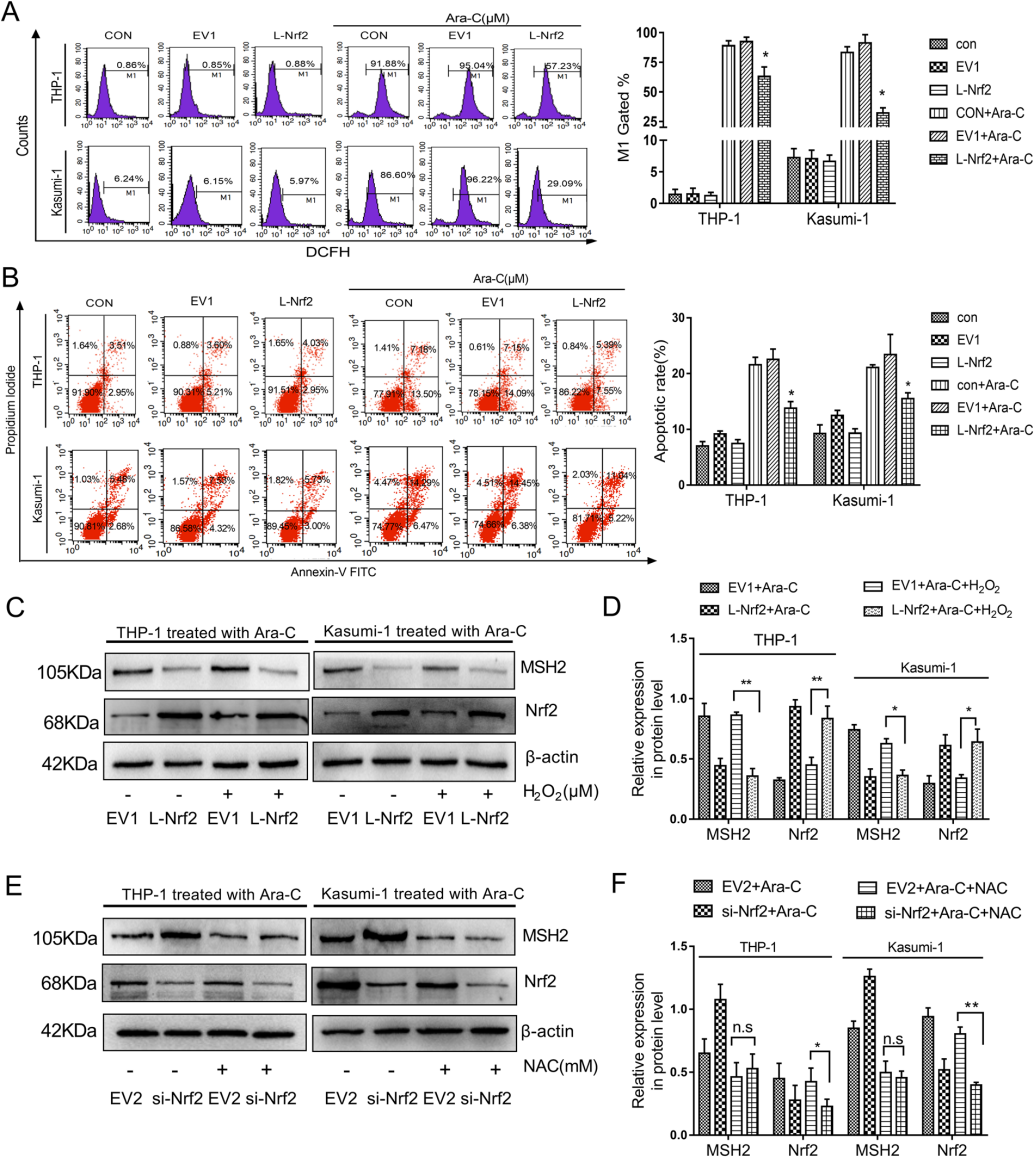
Decrease of ROS generation wasn’t involved to MSH2 downregulation induced by Nrf2. **a** The cells were treated with Ara-C (2 μM) for 24 h. Nrf2-overexpressing and empty vector cells were stained with DCFH-DA to measure intracellular ROS production by flow cytometry. **b** The percentage of apoptotic cells was demonstrated by flow cytometry in both cell lines following the overexpression of Nrf2. **c** Nrf2-overexpressing cells were pretreated with or without H_2_O_2_ (50 μM). Protein expression levels of Nrf2 and MSH2 were detected by western blotting. **d** The relative gray values were shown in histogram. **e** Silencing Nrf2 cells were pretreated with or without NAC (5 mM). Protein expression levels of Nrf2 and MSH2 were detected by western blotting. **f** The relative gray values were shown in histogram. Data are presented as the mean ± SD of three independent experiments. EV, empty vector. Ara-C, cytarabine. NAC, Nacetylcysteine. *P < 0.05, **P < 0.01, ns, no significance.

When tumor cells were stimulated by chemotherapy, Nrf2, as an important transcription factor of antioxidant stress, could significantly inhibit the production of ROS^31^. ROS is also an important factor that initiates the cellular MMR system^32^. Previous research had shown that Nrf2 regulated apoptosis of AML cell lines. Next, the mechanism of DNA MMR induced by Nrf2 in regulating ROS was investigated. We observed ROS levels in these cell lines at 24 h after Ara-C treatment. The results showed that ROS was higher than that before 2 μM Ara-C treatment of cells in each group. However, the elevated ROS in the Nrf2-overexpressing group was significantly lower than that of the EV group and the control group (*P* < 0.05, Fig. 5a). The apoptotic rate was measured by the Annexin V/PI assay after treating Nrf2-overexpressing cells with 2 μM Ara-C for 24 h. The apoptosis of the Nrf2-overexpressing group was significantly decreased in comparison with EV and control group (*P* < 0.05, Fig. 5b).

If Ara-C induced ROS production can account for the increase of MSH2 protein in AML cells, H_2_O_2_ should increase intracellular ROS and elevate MSH2 expression in Nrf2-overexpressing cells. To examine whether there was an intrinsic link between ROS accumulation and the protein levels of MSH2 in AML cells, we pretreated THP-1 and Kasumi-1 cells with H_2_O_2_ (50 μM) for 6 h in Nrf2-overexpressing cells. The results showed that MSH2 expression in the Nrf2 overexpression group pretreated with H_2_O_2_ was still weakened (Fig. 5c, d). In addition, we pretreated the Nrf2 down-regulation group with the ROS scavenger NAC (5 mM) for 2 h and then exposed to Ara-C for 24 h. Western blot assays showed that THP-1 and Kasumi-1 cells treated with Ara-C in combination with NAC weaken MSH2 expression upon Nrf2 knockdown, and there was no significant difference compared with the EV group (Fig. 5e, f). Collectively, these findings revealed that Nrf2 inhibited ROS elevation induced by Ara-C, leading to resistance of AML cells to chemotherapy. However, the ROS level of the Nrf2-overexpressing cells had no significant effect on the MSH2 expression.

### Nrf2 inhibited MSH2 expression in AML cells by activating the JNK/c-Jun signaling pathway

**Fig. 6:**
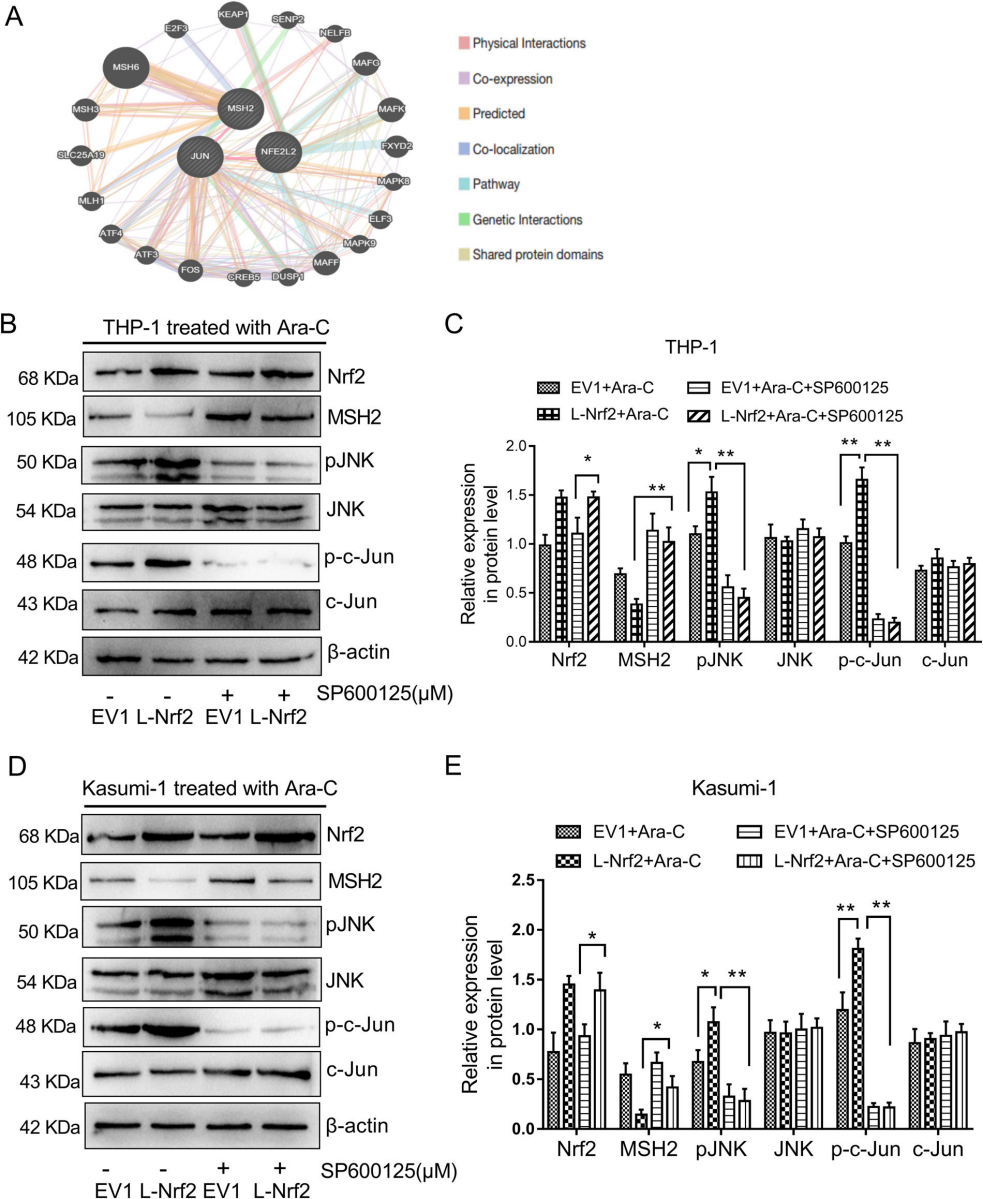
Nrf2 inhibited MSH2 through JNK/c-Jun signaling. **a** Protein-protein interaction network of Nrf2,MSH2 and JUN (GeneMANIA). **b** After treatment with or without 10 μM SP600125 for 24 h in THP-1 cells, protein expression levels of Nrf2, MSH2, JNK, pJNK, c-Jun and p-c-Jun was evaluated by western blot analysis in the Nrf2-overexpression and EV groups. **c** The relative gray values were shown in histogram. **d** After treatment with or without 10 μM SP600125 for 24 h in Kasumi-1 cells, protein expression levels of Nrf2, MSH2, JNK, pJNK, c-Jun, and p-c-Jun was evaluated by western blot analysis in the Nrf2-overexpression and EV groups. **e** The relative gray values were shown in histogram. Data are presented as the mean ± SD of three independent experiments. *P < 0.05, **P < 0.01, ns, no significance.

Based on the above results, we found that the inhibition of MSH2 by Nrf2 was not dependent on the ROS accumulation. To investigate the underlying mechanism, we used GeneMANIA’s PPI network (https://www.oncomine.org/) and revealed the relationship between Nrf2 and MSH2. The results showed that Nrf2 might regulate MSH2 through the JUN signal pathway (Fig. 6a). We quantified the expressions of c-Jun and c-Jun N-terminal kinase (JNK) by western blotting with the Nrf2 overexpression. The results showed that Nrf2 was positively correlated with c-Jun. Nrf2 overexpression in THP-1 and Kasumi-1 cells dramatically increased phosphorylated JNK and c-Jun levels compared to EV cells (Fig. 6b–e). Furthermore, we selected a JNK inhibitor (SP600125) to complete the following experiment. Then, AML cells were treated with 10 μM SP600125 for 24 h. Although the expression of Nrf2 in THP-1 and Kasumi-1 cells changed slightly, the protein levels of pJNK and p-c-Jun were decreased. Conversely, the protein levels of MSH2 were increased in Nrf2-overexpressing cells (Fig. 6b–e). In summary, these data indicated that Nrf2 overexpression inhibited MSH2 expression through activating the JNK/c-Jun signaling pathway.

## Discussion

Chemoresistance is one of the major difficulties during cancer chemotherapy. According to whether the occurrence of drug resistance is related to gene mutation, it can be divided into gene instability-dependent and independent drug resistance. Previous reports in AML, and other solid tumors have shown that Nrf2 is associated with resistance to chemotherapeutic agents^33–35^. The high Nrf2 expression can lead to the gene instability-independent drug resistance in AML, and its mechanism is mostly related to the activation of NF-kB^36^. However, there are few reports about the relationship between Nrf2 and gene instability-dependent chemoresistance. In the current study, we characterized the role of Nrf2 in AML gene instability-dependent chemotherapy resistance and investigated its underlying molecular basis.

According to the whole-exon sequencing analysis, AML patients with high Nrf2 expression had a higher tumor mutation burden. Furthermore, patients in the Nrf2-High group were prone to relapse and chemoresistance. In addition, we evaluated differentially expressed genes in Nrf2-High/Low group and found that high Nrf2 expression significantly inhibited the DNA MMR pathway in AML. MMR plays a crucial role in regulating tumor gene mutation^37^. The abnormal expression of MMR-related proteins is also related to tumor drug resistance^38^. In the case of leukemia, Diouf et al. observed that the protein level of DNA mismatch repair protein MSH2 in 11% of childhood acute lymphoblastic leukemia cells decreased significantly and was resistant to mercaptopurine^39^. According to the research by Mao et al., 34.0% of AML patients had MMR gene mutation or MLH1 promoter methylation, and the incidence of MMR deficiency in refractory or recurrent AML patients was significantly higher than that in newly diagnosed patients^40^. These findings indicate that DNA MMR is crucial in the progression of leukemia. In our study, Nrf2 expression mainly induced gene instability-dependent drug resistance in AML by inhibiting DNA MMR, which was consistent with the above findings.

High Nrf2 expression caused MMR deficiency and increased the tumor mutation burden, whereas its exact roles in AML remain understudied. Here, we provided the significant evidence that Nrf2 overexpression could induce drug resistance in AML by suppressing MSH2. In vitro, Nrf2 overexpression protected the AML cells from apoptosis and suppressed MSH2 in AML cells. In addition, we found that mice bearing AML cells with Nrf2 overexpression demonstrated higher leukemia infiltration, lower survival, and MMR deficiency in vivo. In sum, these results implied that Nrf2 overexpression leads to gene instability-dependent drug resistance by suppressing MSH2 expression.

Understanding the functional mechanism of Nrf2 suppressed DNA MMR in AML will greatly facilitate the development of drug resistance therapy. The continuous activation of Nrf2 leads to the relative decrease of intracellular ROS. A certain concentration of ROS can promote cell growth^41^. And excessive accumulation of ROS can increase the methylation level of MMR-related factors MLH1 and MSH2 promoter, resulting in decreased expression and loss of function^32^. As the upstream regulatory genes of MSH2, mTOR, HERC1, PRKCZ, and PIK3C2B, are mutated, it will lead to a decrease in MSH2 expression and a MMR deficiency state in tumor cells^39^. In this study, after the treatment of the cells with Ara-C, a significant increase in ROS generation was detected by flow cytometry and DCFH-DA in the Nrf2-overexpressing group, while the increasing levels of ROS in the Nrf2-overexpressing group was significantly lower than that in the EV group and control group. To further investigate the effect of intracellular ROS on MSH2 protein expression, we changed ROS levels in THP-1 and Kasumi-1 cells. When NAC was applied, there was no significant difference in MSH2 expression in the Nrf2 silent group compared with the EV group. However, Nrf2 overexpression still suppressed MSH2 expression after Nrf2-overexpressing cells treatment with Ara-C in combination with H_2_O_2_. This finding demonstrates that regulation of MSH2 by Nrf2 is not depended on the ROS signal.

**Fig. 7:**
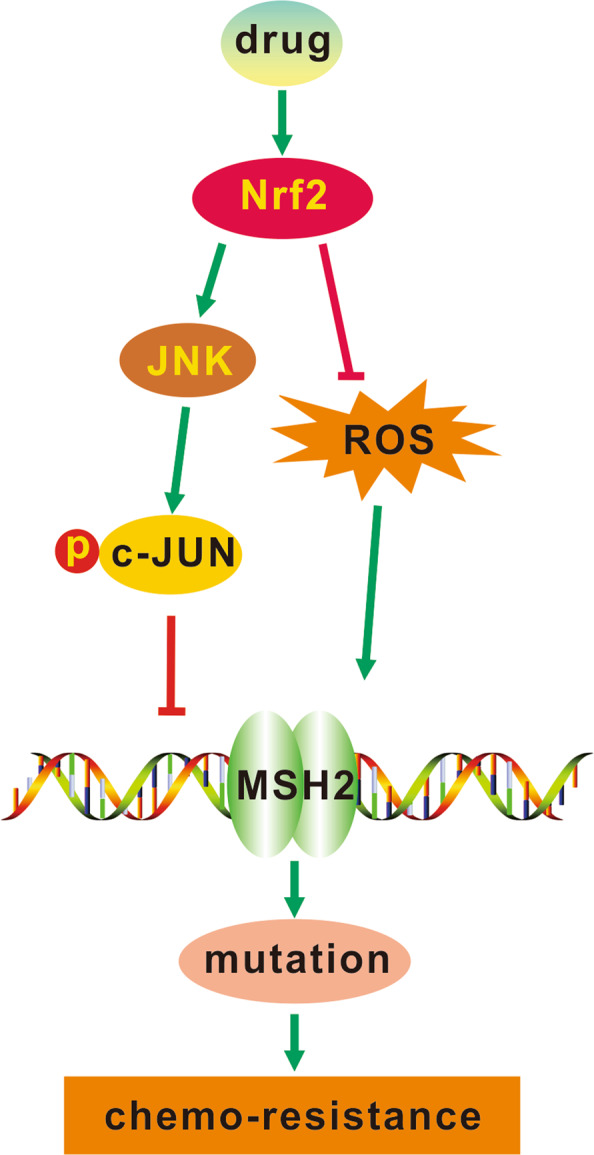
Schematic representation of Nrf2 mediated gene mutation-dependent resistance of AML cells to chemotherapy. Nrf2 reduced cytarabine-induced ROS and positively regulated JNK, activating the phosphorylated c-Jun, leading to inhibition of DNA MMR and finally mutation-dependent chemoresistance.

Our research team had previously proved that overexpression of heme oxygenase-1 (one of Nrf2 target genes) promoted proliferation and increased resistance to Ara-C-induced apoptosis of AML cells in vitro and the leukemia’s progression of AML in vivo by activating the JNK/c-Jun signaling pathway^42^. The c-Jun oncogene is a member of the activator protein-1 (AP-1) family of transcription factors that is phosphorylated and activated by the JNK^43^. A previous study suggested that shRNA-mediated inhibition of Jun decreased AML cell survival and propagation in vivo^44^. These studies demonstrated that JNK/c-Jun activation played an important role in AML. Given the significance of the JNK/c-Jun signaling pathway in AML, our research provided novel insights that Nrf2 inhibited MSH2 expression and promoted AML gene instability-dependent chemoresistance by activating the JNK/c-Jun signaling pathway (Fig. 7).

However, the mechanism underlying the activation of JNK by Nrf2 remains unclear. Notably, upon UV irradiation, a cooperation of p53 and the c-Jun pathway activates transcription of MMR gene MSH2^45^. According to references and our results, the high level of Nrf2 exerts an MMR deficiency effect on tumor cells by the JNK/c-Jun signaling pathway which probably suppresses p53 or up-regulates DUSP1^46,47^, but this postulation still needs further exploration.

In conclusion, our results provide evidence that high Nrf2 expression inhibites MSH2 expression through activating the JNK/c-Jun signaling pathway, playing a key role in the frequency of gene mutation in tumor cells, thus achieving chemoresistance in AML. We propose an underlying regulatory mechanism that Nrf2 induces gene mutation-dependent drug resistance of AML.

## Materials and methods

### Patients’ specimens and cell lines

Using a simple random sampling, we collected 33 bone marrow specimens of AML patients from September 2018 to May 2019 at the Affiliated Hospital of Guizhou Medical University. Details of clinical information are provided in Table 1. Patient samples at diagnosis and relapse were collected, prior to treatment. Prior patient consent and approval from the Institutional Research Ethics Committee were obtained.

**Table 1:** Characteristics of patient samples.

Patients no.	Age (years)	Gender	FAB subtype	Cell count(×10^9^/l)			BM Blast (%)	Karyotype
				WBC	HB	PLT		
1	45	M	M2	30.1	87	9	70.5	46,XX,t(8,21)(q22;q22)
2	41	F	M5	14.58	103	62	60.4	46,XX
3	49	M	M4	4.84	69	100	88	45,XY,-7
4	65	M	M5	220	85	52	86.8	46,XY
5	61	F	M5	4.61	71	197	32.4	46,XX
6	48	F	M5	17.8	57	41	33.9	46,XX
7	51	F	M5	21.17	109	231	90	46,XX
8	54	M	M2	143.19	60	15	92.3	47,XY, + 8
9	29	F	M2	197.12	65	23	71	46,XX
10	21	M	M2	269.13	42	53	26.4	46,XY
11	35	M	M2	31.65	90	330	31	46,XY
12	55	M	M5	88.30	59	17	69	46,XY
13	20	M	M2	6.1	68	12	68	46,XY
14	43	F	M5	0.36	46	15	35.5	46,XX
15	62	M	M2	40.49	99	19	65.7	46,XY
16	79	M	M2	110.18	30	8	49.4	46,XY
17	29	F	M2	41.42	79	10	68.4	46,XX
18	30	F	M2	8.81	106	20	59.5	46,XX
19	49	M	M5	107.14	29	14	47.3	46,XY
20	44	M	M2	179.46	79	64	92.4	46,XY
21	32	F	M5	31.43	75	94	72.1	46,XX
22	48	F	M2	334.80	52	501	60.1	46,XX,t(6;9)(p23;q34)
23	74	M	M2	70.08	50	30	73.3	46,XY
24	40	F	M5	15.4	63	21	33.8	46,XX
25	55	M	M4	42.84	52	56	48.6	46,XY
26	20	F	M2	8.7	125	159	34.4	46,XX
27	49	M	M5	0.35	64	16	49.4	46,XY
28	22	F	M2	142.44	40	29	82.7	46,XX
29	64	F	M2	4.37	52	27	33	46,XX
30	64	F	M5	2.5	72	36	55.8	46,XX
31	21	M	M5	4.01	62	30	47.2	46,XY
32	53	M	M4	30.95	95	15	74.8	46,XY,t(3;5)(p25;q22)
33	74	M	M2	12.86	65	310	51.5	46,XY

*BM* bone marrow, *F* female, *FAB* French–American–British, *HB* hemoglobin, *M* male, *PLT* platelet, *WBC* white blood cell

Human AML cell lines Kasumi-1 and THP-1 were obtained from Guizhou Province Laboratory of Haematopoietic Stem Cell Transplantation Center. All human cell lines were tested for mycoplasma contamination and verified by short tandem repeat profiling. The cell lines were cultured in RPMI-1640 medium supplemented with 10% fetal bovine serum, penicillin (100 units/mL) and streptomycin (100 mg/mL) at 37 °C in a humidified atmosphere with 5% CO_2_.

### Reagents and antibodies

Cytarabine (Ara-C), and SP600125 (a JNK inhibitor) were purchased from MCE, China. N-acetylcysteine (NAC), a reactive oxygen species (ROS) scavenger, was purchased from Coolaber (Beiijng, China). Fetal bovine serum and RPMI 1640 medium were obtained from Gibco (Carlsbad, CA, USA). Western blot analysis was performed using anti-JNK (#9252), anti-phospho-JNK (#4668), anti-phospho-c-Jun (#2361), and anti-c-Jun (#9165) antibodies purchased from Cell Signaling Technology (Danvers, MA, USA). Anti-MSH2 (15520-1-AP) and anti-β-actin (20536-1-AP) antibodies were obtained from Proteintech Group Co., Ltd. (Wuhan, China). Anti-Nrf2 (K106685P) antibody was obtained from Solarbio (Beijing, China).

### Lentiviral transduction

Human Nrf2 overexpression clone lentiviral particle (L-Nrf2) and human Nrf2-RNAi were purchased from Genechem Co., Ltd. (Shanghai, China). Transfection of Nrf2 was performed using the manufacturer’s instructions. Cells (THP-1 and Kasumi-1) respectively transfected with empty vector (EV) were used as controls. After expansion and maintenance in RPMI-1640 medium supplemented with 10% FBS for 5 days, stable THP-1 and Kasumi-1 cell lines expressing L-Nrf2 or si-Nrf2 were selected by puromycin (1.5 μg/ml and 2 μg/ml respectively).

### Quantitative real-time PCR (qRT-PCR)

Total RNAs from cells were extracted using Trizol reagent (Invitrogen, Carlsbad, CA, USA) according to the manufacturer’s instructions. Real-time PCR was performed using the SYBR Green PCR Master Mix (TianGen Biotech, Beijing, China) and the PRISM 7500 real-time PCR detection system (ABI, USA). The qRT-PCR primers (Generay Bioteach Co. Ltd, Shanghai, China) are provided in Table 2.

**Table 2:** The characteristics of the primers used for qRT-PCR.

GENE	Sequence (5′->3′)	Sequence (5′->3′)
β-actin	Forward Primer	GAGACCTTCAACACCCCAGC
	Reverse Primer	ATGTCACGCACG ATTTCCC
MLH1	Forward Primer	TTCTTACTCTTCATCAACCATCGTC
	Reverse Primer	TTCTGGGGACTGATTTCTAAACTGA
MSH2	Forward Primer	GCCAAGAAGTTTCAAA GACAAGC
	Reverse Primer	GGAGAAGTCAGAACGAAGATCAG
MSH6	Forward Primer	TAACGGTTCCTACCAATC
	Reverse Primer	GGGATACAGCCTTTGACC
PMS2	Forward Primer	TTGTGCCCCTGGACTTTTCT
	Reverse Primer	ATCTTCGGCTGCTTG ATTTTCTC
Nrf2	Forward Primer	ACCTCCCTGTTGTTGACTT
	Reverse Primer	CACTTTA TTCTTACCCCTCCT
RFC4	Forward Primer	TTCCAGGTGGTCCGTAAA
	Reverse Primer	CAAGGATCGAGGAGTAGCT

### Western blotting analysis

A BCA protein assay kit (Pierce, Hercules, CA, USA) was used to determine the protein concentrations. Protein (40 μg) was then loaded on 10% SDS–PAGE gel and the separated proteins transferred onto PVDF membranes. Protein blots were incubated with the antibody against the protein of interest. All protein bands were visualized by using an ECL kit (7 Sea Biotech, Shanghai, China).

### Hoechst 33342 staining assay

THP-1 and Kasumi-1 cells were treated with 2 µM of Ara-C for 24 h. Following treatment, the cells were incubated with Hoechst 33342 (10 μg/mL) according to the manufacturer’s instructions (Solarbio, Beijing, China). Nuclear morphological changes were observed under a confocal microscope (Carl Zeiss, Oberkochen, Germany). Normal cell nuclei are homogeneously stained as blue, whereas the nuclei of apoptotic cells display chromatin condensation or nuclear fragmentation. Nuclei were counted from five different areas randomly for the percentage of fragmented nuclei (apoptosis) in each group.

### Apoptosis assay

Apoptosis was determined by double staining of the AnnexinV-FITC and propidium iodide (PI) according to the manufacturer’s instructions (7 Sea Biotech, Shanghai, China). The number of apoptotic cells was measured by flow cytometry using Cell Quest software (BD Biosciences, San Jose, CA, USA).

### ROS Detection

The ROS levels induced by Ara-C in THP-1 and Kasumi-1 cells were detected using the probe 2′, 7′-dichlorodihydrofluorescein diacetate (DCFH-DA, Beyotime, Beijing, China) according to the manufacturer’s instructions and assessed for fluorescence intensity using a flow cytometer.

### Xenografted tumor model

NOD-SCID/IL2Rγc mice were purchased from Model Organisms Center (Shanghai, China). Stably transfected Nrf2 cells were resuspended in PBS at a concentration of 5 × 10^6^ cells/100 μL and then subcutaneously injected into the 5-week-old female mice. The mice were randomly divided into four groups: EV, Nrf2, EV + Ara-C and Nrf2+Ara-C groups (*n* = 4 per group). Once tumors were visible or palpable, mice were treated with Ara-C (60 mg/kg/day for 5 days) by intraperitoneal injection^48^. Mice were placed on the platform of BLT In-Vivo Imaging System (BLT Photon Tech., Guangzhou, China). Tumor weight and diameter were measured every week. All experiments on mice were approved by the Institutional Animal Care and Use Committee of Guizhou Medical University, China. Although there is no blinding in this experiment, it avoids introducing bias in the evaluation of experimental data.

### Immunocytochemical (ICC) and Immunohistochemical (IHC) staining

ICC and IHC staining with antibodies against Nrf2 and MSH2 were performed to detect protein expression levels following standard operating procedures. The positive staining scores were calculated by multiplying the percentage positive (No staining, 0; Pale yellow, 1; Tan, 2; Brown, 3; Nuclear staining in 0–25% of cells, 0; Nuclear staining in 25–50% of cells, 1; Nuclear staining in 50–75% of cells, 3; Nuclear staining in 75–100% of cells, 4).

### Statistical analysis

GraphPad Prism 7.0 software (Graphpad Software, Inc, USA) was used to statistically analyze the data. Clinical data were evaluated by the Shapiro–Wilk normality test for normal distribution. Experimental data obtained from at three separate experiments were presented as mean ± standard deviation (SD). Differences between two groups were analyzed using an unpaired two-tailed Student’s *t* test. The comparison among three or more groups was analyzed using one-way ANOVA. Survival was presented with a Kaplan–Meier survival plot. A *P* value of less than 0.05 was considered statistically significant.

## Supplementary information

Supplementary figure 1

Supplementary figure 2

Supplementary figure legend
