# Supplementary figures and images for: Nrf2 overexpression increases risk of high tumor mutation burden in acute myeloid leukemia by inhibiting MSH2
Source: Cell Death Dis. 2021 Jan 5;12(1):20. doi: 10.1038/s41419-020-03331-x (PMC7790830; doi:10.1038/s41419-020-03331-x)

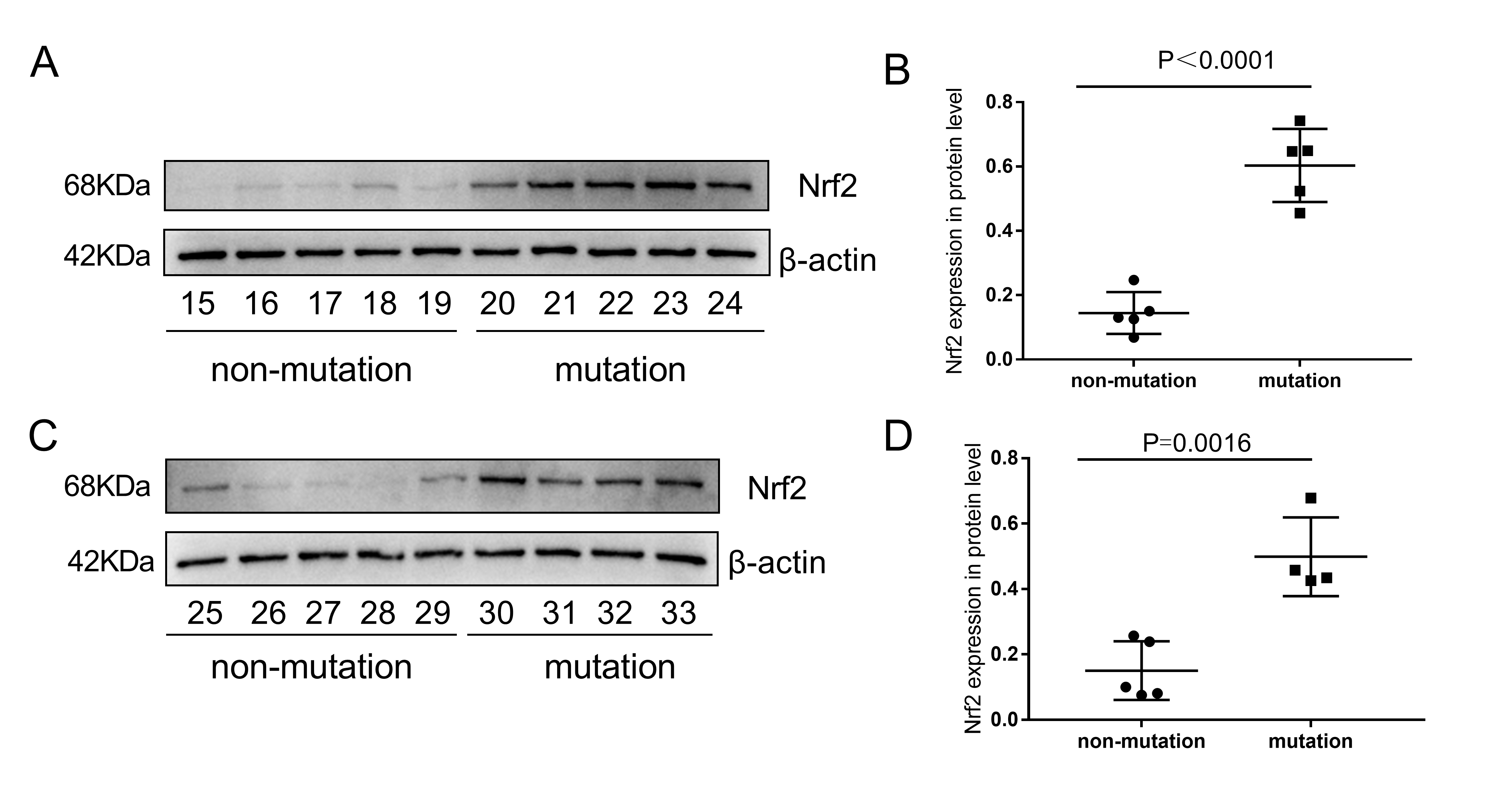

Supplement: Supplementary file 1 — Supplementary figure 1 [file 41419_2020_3331_MOESM1_ESM.tif]

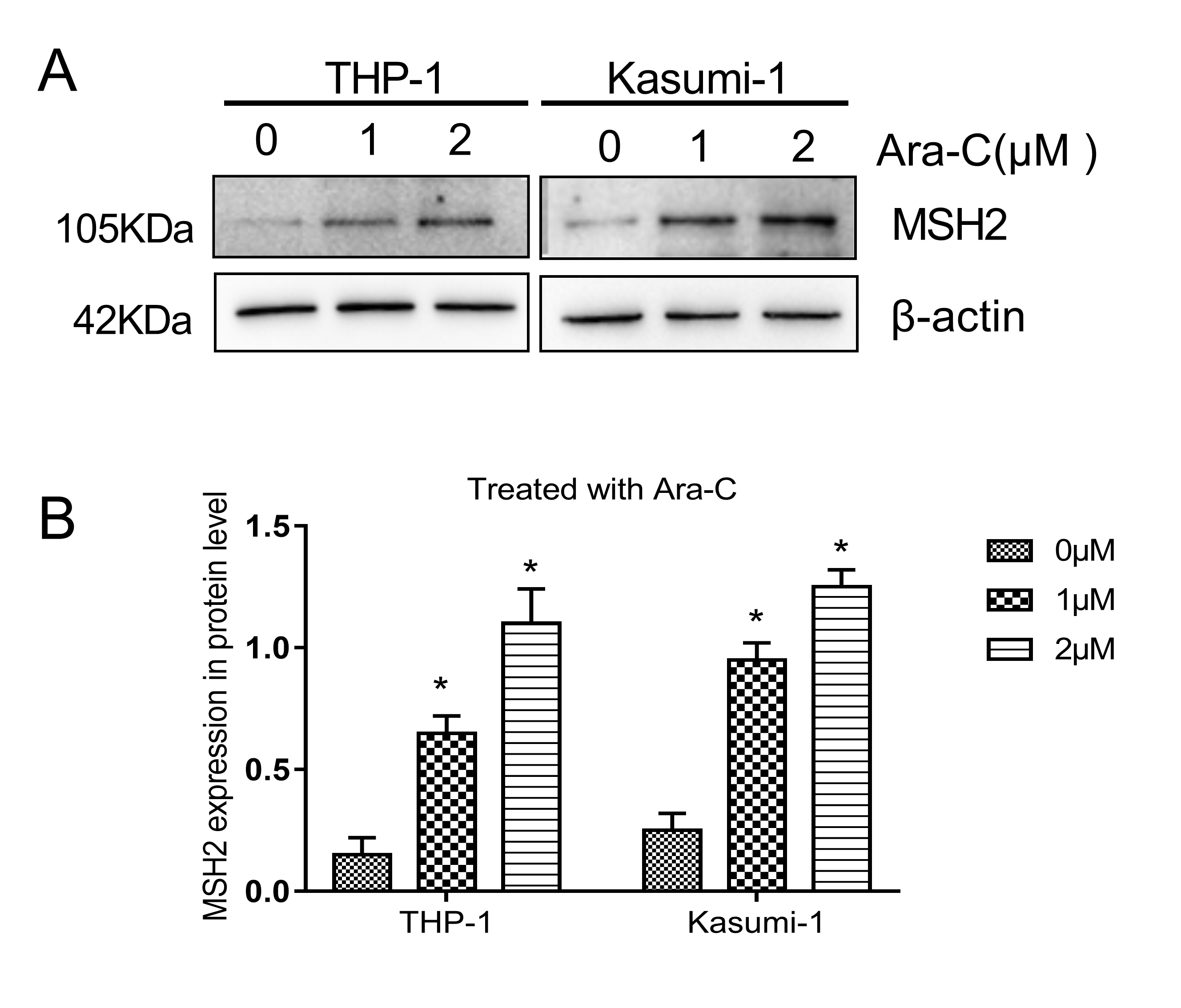

Supplement: Supplementary file 2 — Supplementary figure 2 [file 41419_2020_3331_MOESM2_ESM.tif]
