# Supplementary material for: Nrf2 overexpression increases risk of high tumor mutation burden in acute myeloid leukemia by inhibiting MSH2
Source: Cell Death Dis. 2021 Jan 5;12(1):20. doi: 10.1038/s41419-020-03331-x (PMC7790830; doi:10.1038/s41419-020-03331-x)
Supplement: Supplementary file 3 — Supplementary figure legend [file 41419_2020_3331_MOESM3_ESM.docx]

**Figure S1**. Expression levels of Nrf2 protein were detected in AML samples by western blotting (n=19) and the relative gray values were shown in histogram.

**Figure S2**. The MSH2 protein levels were accumulated during exposure to Ara-C in THP-1 and Kasumi-1 cells assessed by western blot and the relative gray values were shown in histogram.
